# Targeting nontuberculous mycobacteria with phages: optimization of *in vitro* host range assays

**DOI:** 10.1186/s12866-026-05452-3

**Published:** 2026-07-30

**Authors:** Tobias Weirauch, Eva Brügger, Kai Litzius, Maria J. G. T. Vehreschild, Silvia M. Würstle, Simone C. Lieberknecht-Jouy

**Affiliations:** 1 Department II of Internal Medicine, Infectious Diseases, Goethe University Frankfurt, University Hospital Frankfurt, Frankfurt Am Main, Theodor-Stern-Kai 7, Frankfurt, 60590 Germany; 2 Experimental Physics V, Center for Electronic Correlations and Magnetism, University of Augsburg, Augsburg, Germany

**Keywords:** Mycobacteriophages, Nontuberculous mycobacteria, Phage assay, Host range testing, Cultivation comparison

## Abstract

The clinical implementation of mycobacteriophages is hampered by the lack of standardized *in vitro* methods and inconsistent host range results, limiting reliable assessment of their therapeutic potential. Nontuberculous mycobacterial (NTM) infections are frequently difficult to treat, emphasizing the urgent need for alternative or adjunctive strategies to antibiotics. To facilitate reproducible translational research, a detailed and systematic understanding of *in vitro* assay conditions is essential. Here, we comprehensively evaluated commonly used liquid and solid media for their suitability in supporting growth of multiple NTM strains and subsequent phage application. We compared bacterial growth kinetics, aggregation tendencies, and the efficiency of phage-induced lysis, including plaque formation, across three liquid media and corresponding solid agars. Our results demonstrate that Middlebrook-based media and agar consistently support robust NTM growth, reduce bacterial clumping, and enable reproducible detection of phage activity, outperforming TSB-based alternatives despite higher cost and labor requirements. These findings provide a standardized framework for *in vitro* NTM cultivation and phage testing, which is critical for reliable evaluation of host range, infection dynamics, and the development of mycobacteriophage-based therapies.

**Supplementary Information:**

The online version contains supplementary material available at 10.1186/s12866-026-05452-3.

## Introduction

Nontuberculous mycobacteria (NTM) pose a significant risk particularly to patients with structural lung diseases, immunomodulatory therapy, primary immunodeficiencies, or HIV infection [1, 2]. The majority (80–90%) of NTM-associated diseases affect the respiratory system, with the slow-growing *Mycobacterium avium complex* (MAC), rapid-growing *M. abscessus* complex, and *M. kansasii* accounting for most cases [3]. Treatment is challenging due to inherent and acquired antibiotic resistance and potential toxicity from prolonged drug regimens [4, 5], highlighting the urgent need for safe adjunctive therapies.

Bacteriophages offer a promising complementary approach [6–8]. However, the limited repertoire of therapeutically useful phages and the high genetic diversity of some NTM strains necessitate further *in vitro* studies. Despite the increasing number of case reports and phage discovery papers, standardized and universally accepted *in vitro* phage assay protocols are still lacking. Current literature often relies on diverse technical approaches, where critical parameters, such as precise media composition, the choice between Top Agar (TA) and Direct Plating (DP) formats, or the definition of growth phases, vary significantly or are under-described [9–13]. These inconsistencies complicate the translation of research findings into reproducible clinical workflows, particularly for patient-specific phage susceptibility testing.

Reliable assessment of phage efficacy requires a systematic quantification of how different cultivation standards and evaluation methods influence experimental outcomes. For instance, while Middlebrook media supplemented with oleic acid-albumin-dextrose-catalase (OADC) are widely used in NTM research, and phage studies often adopt these media [9–12], their high cost and complexity may not be optimal for all diagnostic settings. Alternative approaches, such as tryptic soy broth (TSB)-based media have been described but rarely benchmarked against traditional standards regarding their impact on plaque visibility or growth kinetics [13].

This study addresses these gaps by evaluating the central question: Among commonly used media and plating formats, which conditions provide the most robust and sensitive detection of phage activity across representative NTM strains, and how do these trade off against practical constraints such as cost and preparation time? Our goal is to provide a validated framework for routine susceptibility testing rather than initial high-throughput screening. Specifically, we aim to (i) provide detailed NTM cultivation protocols for *in vitro* phage assays, (ii) describe the advantages and limitations of each approach regarding bacterial clumping and physiological relevance, and (iii) compare media and methods in terms of host range, growth kinetics, reproducibility, and cost-efficiency.

## Methods

### Bacterial strains and cultivation

Growth curves were conducted using six mycobacterial isolates. Specifically, we used three isolates of the *Mycobacterium avium* complex (MAC) (*M. avium* subsp. *avium* (53,202,766*)*, *M. chimaera (53,199,666)*, and *M. avium* subsp. *intracellulare* (53,157,420*)*), two isolates of *Mycobacterium abscessus* complex (MABC) (*M. abscessus* subsp. *abscessus (Lab MCT 07)*, *M. abscessus* subsp. *bolletii* (DSM 45149)), and one *M. smegmatis* (040825) isolate. For subsequent experiments, *M. abscessus* subsp. *massiliense* (52,344,518) was additionally included. Most isolates were provided by the Institute of Microbiology, Goethe University Hospital, Frankfurt, Germany. *M. abscessus* subsp*. bolletii* was provided from the Leibniz Institute DSMZ-German Collection of Microorganisms and Cell Cultures GmbH and *M. abscessus* subsp*. abscessus* was provided by the Laboratory for Molecular and Cellular Technology, Queen Astrid Military Hospital, Brussels, Belgium. The panel was deliberately curated to reflect the phylogenetic and phenotypic diversity of clinically relevant NTM, encompassing both slow-growing and fast-growing species to capture key differences relevant to phage-host interactions and assay performance. Unless otherwise stated, all isolates were of clinical origin. For detailed information regarding isolate identifiers, clinical origin, and specific applications, see Table S1.

All strains were initially cultured on Middlebrook/OADC agar at 37 °C. Experiments were conducted within seven days of incubation to ensure high bacterial viability. Cultures that could not be sub-cultivated were considered non-viable and excluded from further analysis.

### Phages and host range determination

Mycobacteriophages 8UZl and D29 were stored at 4 °C in phosphate-buffered saline (PBS). Initial titers of both phages were 10^10^ plaque-forming units (PFU)/mL. Their titers remained stable throughout the experimental period. Host range was determined in advance: 8UZl lysed all above-mentioned mycobacterial isolates, whereas D29 infected only *M. smegmatis*, *M. abscessus* subsp*. abscessus*.

### Experimental replicates

All growth curves were performed with a minimum of four biological replicates. Samples showing no detectable growth or evidence of contamination (implausibly high proliferation within 96 h) were excluded. Clumping assays included four biological replicates, each with three technical replicates. Spot assays were conducted with six biological replicates, each with two technical replicates.

### Culture conditions in liquid media

**Fig. 1:**
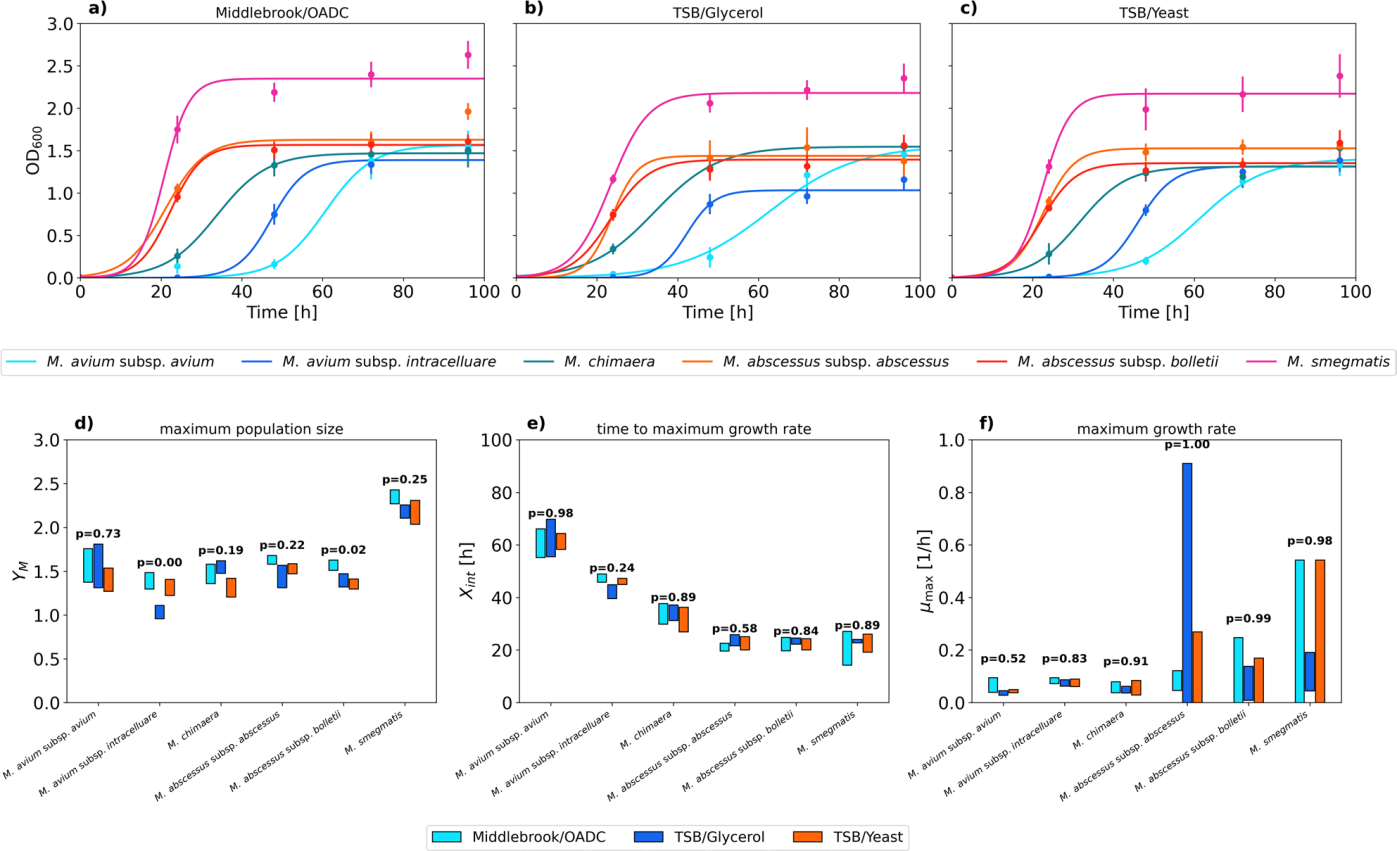
Logistic growth curves (OD_600_) of *M. avium* subsp. *avium*, *M. avium* subsp. *intracellulare*, *M. chimaera*, *M. abscessus* subsp. *abscessus*, *M. abscessus* subsp. *bolletii*, and *M. smegmatis* cultivated in liquid Middlebrook/OADC, TSB/glycerol, or TSB/yeast-based medium are shown over time (**a**-**c**). Fitted maximum OD_600_ values (Y_M_), i.e. a measure for the maximum population size, and fitted growth time to maximum growth rate (X_int_) as well as maximum growth rate µ_max_ with box height corresponding to standard error around the mean for each species and medium (**d**-**f**). *p*-values indicate statistical comparisons via survival function between media for each species

To identify the most appropriate liquid cultivation method, three media were compared: Middlebrook/OADC, TSB/Glycerol, and TSB/Yeast (see Table 1 for full composition). Cultures were grown in 50 mL conical centrifuge tubes (Thermo Fisher Scientific, Waltham, Massachusetts, USA). To standardize starting concentrations, the culture was adjusted to an optical density at 600nm (OD₆₀₀) of 0.5 in 0.5 mL medium before transfer to a conical centrifuge tube. Incubation was performed at 37 °C on a shaking incubator (300 rpm), with bacterial growth monitored photometrically. Triplicates of a dilution series (10⁻^1^ to 10⁻⁸) were spotted on the corresponding agar (1.5%) to calculate CFU. Growth in each medium and agar was compared to determine the logarithmic growth phase (Fig. 1).

**Table 1:** Detailed overview of culture media ingredients

Middlebrook/OADC Medium	TSB/Glycerol Medium	TSB/Yeast Medium
- 2,35 g Middlebrook 7H9 broth base - 450 mL ddH_2_O - 1 mL Glycerol 100% - 5 mL of 0,1 M CaCl_2_ - 5 mL of 0,1 M MgSO_4_ - 50 mL OADC	- 15 g TSB broth base - 500 mL ddH_2_O - 2,5 mL Glycerol 100% - 5 mL of 0,1 M CaCl_2_ - 5 mL of 0,1 M MgSO_4_	- 15 g TSB broth base - 500 mL ddH_2_O - 1,5 mL Yeast extract - 5 mL of 0,1 M CaCl_2_ - 5 mL of 0,1 M MgSO_4_

Top agar Middlebrook/OADC	Top agar TSB/Glycerol	Top agar TSB/Yeast
- 50% Middlebrook/OADC-Media - 50% MBTA-Medium MBTA-Medium - 2,35 g Middlebrook 7H9 broth base - 4,0 g Agar Agar - 500 mL ddH_2_O	- 15 g TSB broth base - 1,75 g Agar Agar - 500 mL ddH_2_O - 2,5 mL Glycerol 100% - 5 mL of 0,1 M CaCl_2_ - 5 mL of 0,1 M MgSO_4_	- 15 g TSB broth base - 1,75 g Agar Agar - 500 mL ddH_2_O - 1,5 mL Yeast extract - 5 mL of 0,1 M CaCl_2_ - 5 mL of 0,1 M MgSO_4_

Bottom agar Middlebrook/OADC	Bottom agar TSB/Glycerol	Bottom agar TSB/Yeast
- 9,5 g Middlebrook 7H10 agar - 500 mL ddH_2_O - 2,5 mL Glycerol 100% - 50 mL OADC enrichment - 5 mL of 0,1 M CaCl_2_ - 5 mL of 0,1 M MgSO_4_	- 15 g TSB broth base - 7,5 g Agar Agar - 500 mL ddH_2_O - 2,5 mL Glycerol 100% - 5 mL of 0,1 M CaCl_2_ - 5 mL of 0,1 M MgSO_4_	- 15 g TSB broth base - 7,5 g Agar Agar - 500 mL ddH_2_O - 1,5 mL Yeast extract - 5 mL of 0,1 M CaCl_2_ - 5 mL of 0,1 M MgSO_4_

**Fig. 2:**
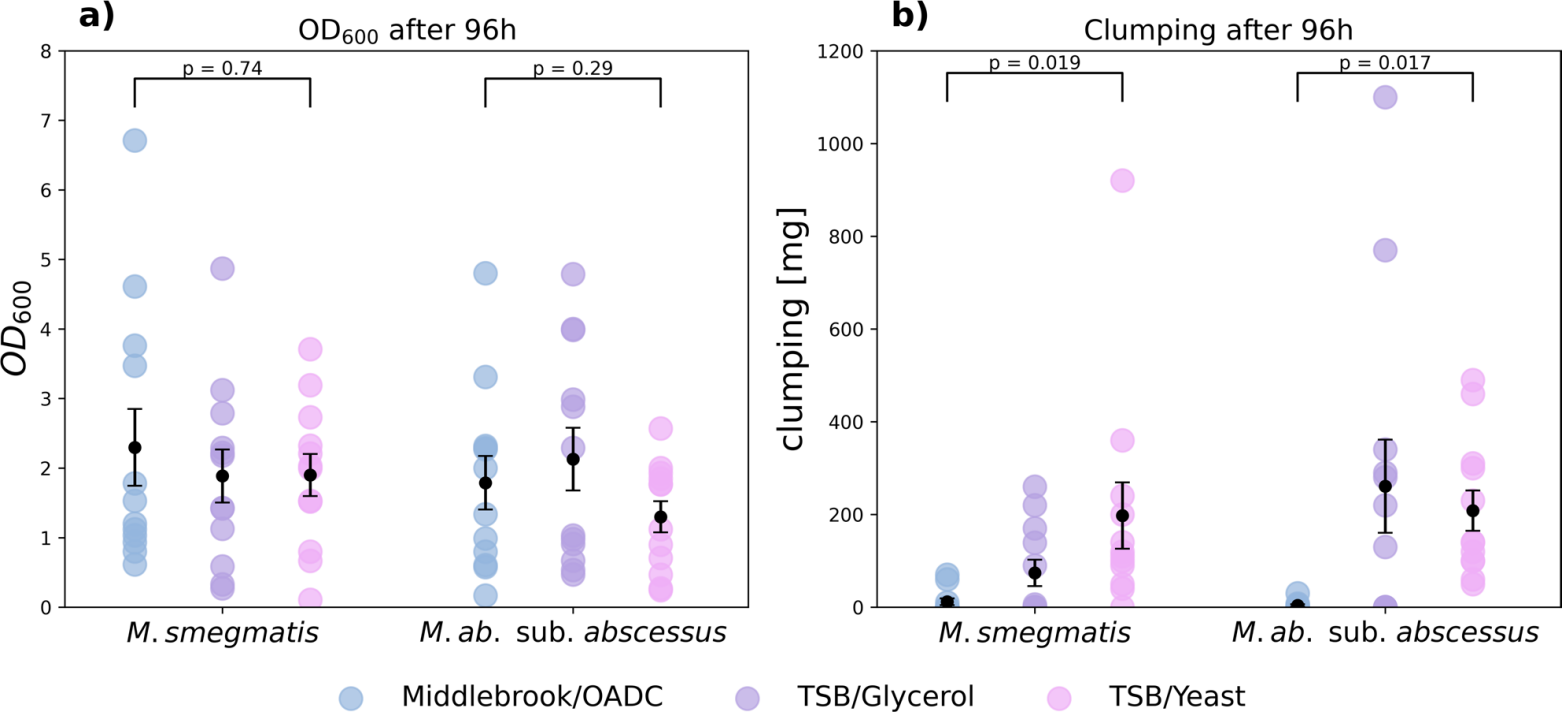
**a** OD_600_; and **b** comparison of bacterial clumping (measured in mg) in *M. smegmatis* and *M. abscessus* subsp. *abscessus* after 96 h of incubation. Four biological replicates, each with three technical replicates, were analyzed for each medium described above. Colored dots represent individual data points; black dots and error bars indicate the mean and standard error of the mean. The indicated p-values correspond to a one-way ANOVA *p*-value

To mitigate the bias in spectrophotometric growth analyses caused by multicellular aggregates and to ensure reproducible multiplicity of infection (MOI)-controlled infections, we implemented a filtration-based method to estimate the degree of clumping across different media conditions. For clumping analysis, *M. smegmatis* and *M. abscessus* subsp*. abscessus* cultures were filtered after 96 h to compare the weight of bacterial aggregates (Fig. 2).

### Comparative phage assays across solid media

In the second phase, we examined phage plaque/lysis appearance (time to positivity) on three agar types: Middlebrook/OADC, TSB/Glycerol, and TSB/Yeast (see Table 1 for detailed manufacturer information). Our modified Middlebrook/OADC and TSB/Yeast protocols were adapted from the Actinobacteriophage Database workflow and the DSMZ instructions for MIDDLEBROOK MEDIUM and TRYPTICASE SOY YEAST EXTRACT MEDIUM [14–16]. The TSB/Glycerol procedure was modified from a protocol described by Pirnay et al. [17].

Two plating approaches were evaluated: the top agar (TA), also referred to as double agar overlay (DAO) method, and direct plating using bacteria embedded in agar. For the TA/DAO method, 15 mL of bottom agar was first poured and allowed to solidify. Subsequently, 4.5 mL of 0.5% top agar was supplemented with 0.5 mL of bacterial culture adjusted to an OD₆₀₀ of 0.5 and overlaid onto the bottom agar. After solidification and drying of the top agar, phage dilution series were spotted in duplicate onto the bacterial lawn. Comparable protocols have been reported previously [18].

For the direct plating approach, 1.1 mL of bacterial culture (OD₆₀₀ = 0.5) was added to 5 mL of medium, mixed with 6 mL of agar, and poured directly onto plates. Therefore, this approach relies on a single agar layer. Similar experimental approaches have been described in previous studies [10, 19].

Bacteriophage application was performed via spot assay, using a dilution series from 10⁻^1^ to 10⁻^10^. Experiments included phage 8UZl across six NTM species and phage D29 on the two susceptible NTM species. Costs of each method were compared (Table 2). Serial dilutions were performed using the same buffer (PBS) across all conditions up to 10^–10^; the consistent absence of lysis at the highest dilutions (i.e., lowest phage concentrations) served as an internal negative control, indicating that the observed lysis at lower dilutions was attributable to phage activity rather than buffer effects.

**Table 2:** Main equipment required for the different methods, together with estimated costs (exchange rate: 1 EUR = 1.13 USD). All listed webpages and products were accessed on May 2, 2025. Individual components may also be available from other suppliers at varying prices

	**Medium**	**Agar**	**Additives**	**Total costs (EUR/USD)**	**Costs per 1000 plates**
Middlebrook/OADC	Middlebrook 7H9 Bouillon-Base 500 g – Merck KGaA Darmstadt, Germany 368,00€; available at: https://www.sigmaaldrich.com/DE/de/product/sial/m0178	Middlebrook 7H10 Agar-Base 500 g—Merck KGaA Darmstadt, Germany 368,00€; available at: https://www.sigmaaldrich.com/DE/de/product/sial/m0303	Middlebrook OADC Growth-Supplement 50 ml – Merck KGaA Darmstadt, Germany 265,00€; available at: https://www.sigmaaldrich.com/DE/de/product/sial/m0678 Glycerin 99.5% 1L —Carl Roth GmbH + Co. KG Karlsruhe, Germany 66,90€; available at: https://www.carlroth.com/de/de/kryokonservierung/glycerin/p/3783.1	1067,90€/$1209,33	28,5 g 7H10 = 20,98€ + 150 ml OADC = 795,00€ + 7,5 ml Glycerin = 0,50€ = 816,48€/$923,22
TSB/Glycerol	BD Bacto™ Tryptic Soy Broth 500 g – Thermo fisher scientific, Waltham, Massachusetts, USA 181,00€; avaialbe at: https://www.fishersci.de/shop/products/bd-difco-dehydrated-culture-media-tryptic-soy-broth-soybean-casein-digest-medium-3/10098983	BD Bacto™ Tryptic Soy Broth 500 g – Thermo fisher scientific, Waltham, Massachusetts, USA 181,00€; avaialbe at: https://www.fishersci.de/shop/products/bd-difco-dehydrated-culture-media-tryptic-soy-broth-soybean-casein-digest-medium-3/10098983 Agar Agar 500 g—Carl Roth GmbH + Co. KG Karlsruhe, Germany 232,50€; available at: https://www.carlroth.com/de/de/agar/agar-agar-bakteriologisch/p/2266.3	Glycerin 99.5% 1L —Carl Roth GmbH + Co. KG Karlsruhe, Germany 66,90€; available at: https://www.carlroth.com/de/de/kryokonservierung/glycerin/p/3783.1	480,40€/$544,02 (Tryptic Soy Broth was only included once in calculation)	45 g TSB = 16,29€ + 22,5 g Agar Agar = 10,46€ + 7,5 ml Glycerin = 0,50€ = 27,25€/$30,81
TSB/Yeast	BD Bacto™ Tryptic Soy Broth 500 g – Thermo fisher scientific, Waltham, Massachusetts, USA 181,00€; avaialbe at: https://www.fishersci.de/shop/products/bd-difco-dehydrated-culture-media-tryptic-soy-broth-soybean-casein-digest-medium-3/10098983	BD Bacto™ Tryptic Soy Broth 500 g – Thermo fisher scientific, Waltham, Massachusetts, USA 181,00€; avaialbe at: https://www.fishersci.de/shop/products/bd-difco-dehydrated-culture-media-tryptic-soy-broth-soybean-casein-digest-medium-3/10098983 Agar Agar 500 g—Carl Roth GmbH + Co. KG Karlsruhe, Germany 232,50€; available at: https://www.carlroth.com/de/de/agar/agar-agar-bakteriologisch/p/2266.3	Yeast extract 250 g—Merck KGaA Darmstadt, Germany 83,30€; available at: https://www.sigmaaldrich.com/DE/de/product/sigma/y1625	496,80€/$562,6 (Tryptic Soy Broth was only included once in calculation)	45 g TSB = 16,29€ + 22,5 g Agar Agar = 10,46€ + 4,5 g Yeast extract = 1,50€ = 28,25€/$31,94

### Statistical analysis

Data were analyzed using jupyter (python) with open-source modules (numpy, matplotlib, skimage, PIL) for fitting and plotting. Logistical growth was fitted as function of time T via

$$f\left(T\right)=\frac{{Y}_{M}}{\mathrm{exp}\left(k \left(-T+{X}_{int}\right)\right)+1}$$

with maximum OD_600_ values (Y_M_), growth speed (k) and growth times (X_int_) under consideration of the error bars (Fig. 1a-c). *p*-value analyses were performed via scipy as indicated in the respective figure caption.

The maximum growth rate ${\mu}_{\mathrm{m}\mathrm{a}\mathrm{x}}=\mathrm{max}\left\{\frac{d}{dT}f\left(T\right)\right\}= \frac{{Y}_{M} k}{4}$ was extracted from the fitted data with error propagation ${\Delta \mu }_{\mathrm{m}\mathrm{a}\mathrm{x}}=\frac{1}{4}\sqrt{{\left({Y}_{\mathrm{M}}\Delta k\right)}^{2}+{\left(k\Delta {Y}_{\mathrm{M}}\right)}^{2}}$. This rate occurs at the inflection point $T={X}_{\mathrm{i}\mathrm{n}\mathrm{t}}$.

Detailed information on the statistical analyses has been deposited on Zenodo (open-access research repository) and can be accessed via the following https://doi.org/10.5281/zenodo.18737049. All procedures were conducted in accordance with relevant guidelines and regulations.

## Results

### Comparative growth kinetics under different liquid culture conditions

Growth dynamics differed between mycobacterial species and culture conditions. In Middlebrook/OADC, all species exhibited logistic growth with species-specific lag phases and plateau levels. *M. smegmatis* showed the fastest growth and highest maximal OD_600_, whereas *M. avium* subsp. avium and *M. avium* subsp. *intracellulare* displayed prolonged lag phases and lower maximal optical densities.

Comparable overall growth patterns were observed in TSB/Glycerol- and TSB/Yeast-based media, although growth kinetics and final OD_600_ values varied between species. Yeast-based medium supported robust growth of *M. smegmatis* and both *M. abscessus* subspecies, while slower-growing isolates exhibited extended lag phases.

Fitted maximum OD_600_ values (Y_M_) were largely comparable across all media for most species (Fig. 1d). No statistically significant differences between media were observed for *M. avium* subsp. *avium* (*p* = 0.73), *M. chimaera* (*p* = 0.19), *M. abscessus* subsp. *abscessus* (*p* = 0.22), or *M. smegmatis* (*p* = 0.25). In contrast, *M. avium* subsp*. intracellulare* displayed a significant difference in Y_M_ between media (*p* < 0.01), with lower fitted maximal OD_600_ values under TSB/Glycerol cultivation.

Analysis of fitted growth times (X_int_) revealed pronounced species-specific differences but no significant media-dependent effects (Fig. 1e). Growth times did not differ significantly between media for *M. avium* subsp. *avium* (*p* = 0.98), *M. avium* subsp*. intracellulare* (*p* = 0.24), *M. chimaera* (*p* = 0.89), *M. abscessus* subsp. *abscessus* (*p* = 0.58), *M. abscessus* subsp*. bolletii* (*p* = 0.84), or *M. smegmatis* (*p* = 0.89). Across all media, slow-growing species exhibited expected longer fitted growth times compared with rapidly growing species.

Maximum growth rates (μ_max_) showed substantial interspecies variability, with rapidly growing species exhibiting consistently higher fitted growth rates than slow-growing species across all media conditions (Fig. 1f). However, no statistically significant media-dependent differences in μ_max_ were detected for any species, including *M. avium* subsp. *avium* (*p* = 0.52), *M. avium* subsp. *intracellulare* (*p* = 0.83), *M. chimaera* (*p* = 0.91), *M. abscessus* subsp*. bolletii* (*p* = 0.99), and *M. smegmatis* (*p* = 0.98). Notably, *M. abscessus* subsp. *abscessus* displayed particularly high fitted μ_max_ values under TSB/Glycerol conditions, although this effect was not statistically supported (*p* = 1.00). In general, μ_max_ provides an intuitive descriptor of the exponential growth behavior and allows discrimination between slow- and fast-growing mycobacterial species. However, compared with Y_M_ and X_int_, μ_max_ represents a less robust fitting parameter, as its estimation is strongly influenced by the temporal resolution and exact positioning of sampling points during the exponential growth phase. Consequently, variations in measurement timing can disproportionately affect fitted μ_max_ values, limiting the statistical interpretability of subtle differences between cultivation conditions.

### Bacterial clumping behavior

Clumping was investigated in *M. smegmatis* and *M. abscessus* subsp. *abscessus*, as bacterial aggregates in both cultures could not be dispersed by vortexing. Four biological replicates with three technical replicates each (12 measurements in total) were analyzed. After 96 h, bacterial clumping in *M. smegmatis* cultures was significantly lower in Middlebrook/OADC medium compared with TSB/Glycerol or TSB/Yeast (*p* = 0.019). Similarly, less clumping was observed in *M. abscessus* subsp. *abscessus* cultured in Middlebrook/OADC compared with TSB–based media (*p* = 0.017). Although bacterial aggregation was lower in Middlebrook/OADC, no significant difference in OD600 was observed (*M. smegmatis*: *p* = 0.73/*M. abscessus* subsp. *abscessus*: *p* = 0.29).

### Method comparison for spot assays

#### Overview of successful and unsuccessful approaches for phage 8UZL

**Fig. 3:**
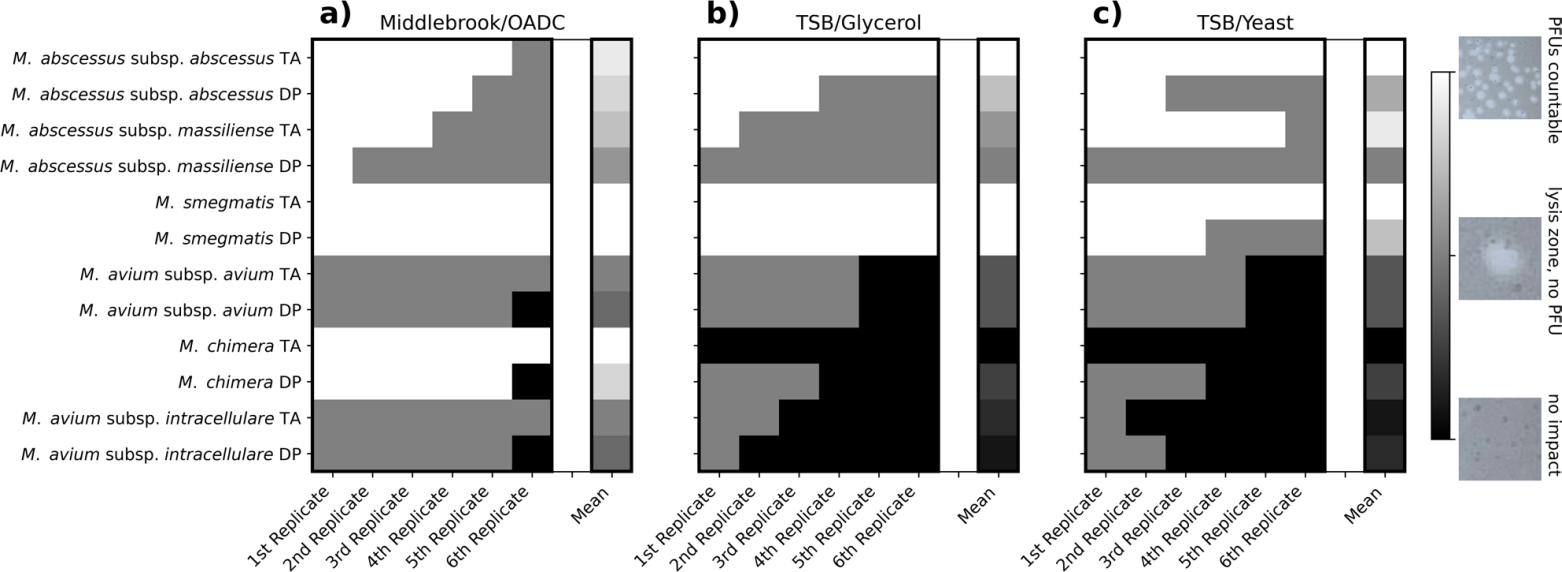
Distribution of phage lytic activity in spot assays on successfully cultivated bacteria. White boxes indicate lysis zones induced by phage 8UZl in the bacterial lawn PLUS countable plaques at any lower dilution between 10^–2^ and 10^–10^ (initial titer of 8UZl = 10^10^ PFU/mL); grey boxes represent phage-induced lysis zones without countable plaques at the terminal dilution; black boxes indicate no detectable phage lytic activity. Assays were performed using six biological replicates, each containing two technical replicates. A mean result is given for each species and growth medium, facilitating comparisons. TA = top agar method (= double agar overly method); DP = direct plating method

A categorical heat map was generated to systematically compare phage lytic activity across diverse solid media and assay formats (TA versus DP), summarizing the outcomes of spot assays performed with successfully cultivated mycobacterial isolates (Fig. 3). Phage activity was classified into three categories: productive infection with countable plaques (PFU), phage-induced lysis without plaque formation at the terminal dilution (lysis zone), and absence of detectable phage activity.

Overall, Middlebrook/OADC-based assays demonstrated the highest proportion of productive phage infections, as indicated by a greater frequency of countable plaques compared with TSB/Glycerol and TSB/Yeast. In addition, only three cultures showed no detectable phage activity under Middlebrook/OADC conditions, whereas a substantially higher number of cultures lacked detectable lysis when cultivated in TSB/Glycerol or TSB/Yeast. These findings indicate a higher robustness and reproducibility of plaque formation using Middlebrook/OADC-based procedures.

For Middlebrook/OADC, TSB/Glycerol, and TSB/Yeast, the TA approach consistently resulted in a higher number of conditions yielding countable plaques. In contrast, direct plating more frequently produced lysis zones without plaque formation or no detectable phage activity. An exception to this general pattern was observed for *M. chimera* cultivated in TSB/Glycerol and TSB/Yeast, where direct plating resulted in a higher frequency of productive plaque formation compared to the TA method.

Taken together, the results highlight pronounced differences in the detection of phage lytic activity depending on both the culture medium and the assay format.

#### Titer-dependent detection of lytic activity of phage 8UZl

To compare the titer-dependent lytic activity of phage 8UZl across culture conditions and assay formats, the range of dilutions producing detectable lysis on bacterial lawns was assessed for each isolate and medium (Fig. S1). Detectable phage activity was defined as the presence of lysis zones and/or countable plaques.

Across all three media, the lowest titer producing detectable phage activity differed between the TA and DP approaches (Fig. S1a, b). In general, experiments performed using the TA method were more sensitive compared to DP, which more frequently resulted in narrower detection windows or loss of detectable lysis at higher dilutions.

When comparing media within the same assay format, no consistent differences in overall sensitivity were observed between Middlebrook/OADC, TSB/Glycerol, and TSB/Yeast. However, differences were observed in the ability of 8UZl to lyse the bacterial lawn of *M. chimera* at lower dilutions depending on the medium. In Middlebrook/OADC, lytic activity extended to lower dilutions compared with TSB-Glycerol and TSB–Yeast.

Together, these data indicate that while the choice of medium does not markedly alter the overall dilution range of detectable phage activity, the assay format affects the dilution-dependent manifestation of lysis on bacterial lawns.

#### Visualization of countable plaques across different conditions for phage 8UZl

**Fig. 4:**
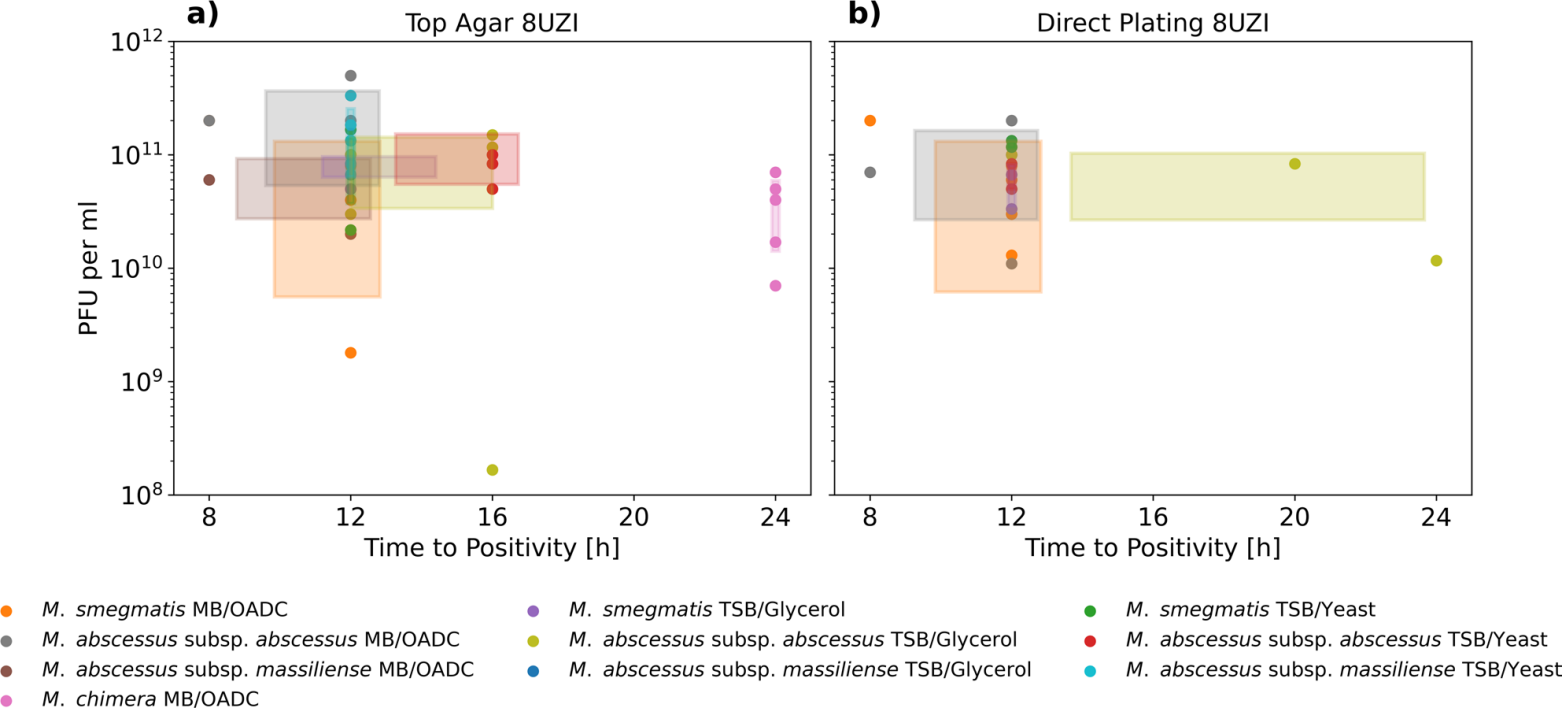
A selection of results in which 8UZ1 produced countable plaques, allowing calculation of PFU, is shown: (**a**) TA results; and (**b**) DP results. Note that the bacterial color scheme does not correspond to the images above, owing to the diversity of data points presented. Dots correspond to individual data points, colored boxes to mean (center of box) in time and PFU/ml as well as their standard deviation (width and height of box). Bigger boxes indicate stronger scattering of the data

Figure 4 presents a quantitative subset of conditions in which phage 8UZl produced countable plaques, allowing calculation of plaque-forming units (PFU), and relates PFU output to the time to positivity (first visual detection of bacterial growth combined with phage lysis at any dilution) across assay formats. Considerable variability was observed both in PFU per milliliter and in the time required for the first detectable phage-induced effect, underscoring the strong influence of bacterial host species and experimental setup on measurable phage activity.

The top agar method enabled plaque detection in a broader range of NTM species than direct plating (Fig. 4a, b). Earlier and more frequent detection was associated with a narrower distribution of PFU values, consistent with more synchronous infection dynamics and improved reproducibility of plaque development under top agar conditions. In contrast, direct plating frequently exhibited delayed positivity and a broader spread of PFU values, suggesting increased variability in phage-host interactions at the bacterial surface.

Despite differences in time to positivity, total PFU counts were comparable between top agar and direct plating for strains in which both methods yielded countable plaques. This indicates that the assay format primarily affected the kinetics of detectable phage activity rather than the overall replication capacity of phage 8UZl.

Together, these results demonstrate that while both assay formats can support productive infection by phage 8UZl, the top agar method provides faster and more consistent readouts, particularly for rapidly growing mycobacterial hosts. Figure 4 therefore complements the categorical analysis presented in Fig. 3 by quantitatively illustrating how assay format and host species jointly shape the dynamics and detectability of phage infection.

### Validation in rapidly growing mycobacteria using phage D29

The experiments were repeated with phage D29 to independently validate the results obtained with 8UZL. However, due to its narrower host range, the analysis was restricted to *Mycobacterium smegmatis* and *Mycobacterium abscessus* subsp. *abscessus*. Consequently, only those rapidly growing mycobacteria could be included for comparative analysis. Results are summarized in Figure S2 (see Supplementary Material) and largely corroborate the findings presented in Figs. 3 and 4.

## Discussion

This work presents three detailed protocols for the cultivation of NTM for *in vitro* mycobacteriophage applications and systematically evaluates their respective advantages and limitations. Beyond protocol descriptions, we propose criteria to guide the choice between top agar and direct plating approaches, highlight critical experimental pitfalls (e.g., bacterial clumping and lysis without countable plaques), and summarize method-specific costs.

Consistent with previous reports, *M. abscessus* spp. and *M. smegmatis* exhibited faster growth across all tested media compared with members of MAC [20–22]. Middlebrook media, such as 7H9 supplemented with OADC, are widely regarded as standard for NTM cultivation and are frequently included in comparative studies [23]. To our knowledge, however, no direct comparison of the three media evaluated here has previously been reported. Our bacterial growth curve analyses demonstrate that all tested media support liquid culture incubation of the tested isolates, indicating that expensive supplements such as OADC are not strictly required during this stage of the workflow. Although cultures were OD600-standardized, the slow growth and inherent clumping of NTM can compromise the accuracy of OD₆₀₀ as a proxy for cell density. Consequently, some cultures may have entered exponential growth earlier or exceeded the target OD₆₀₀, reflecting intrinsic variability in growth kinetics.

In contrast, bacterial aggregation differed markedly between media. Greater clumping was consistently observed in TSB-based media compared with Middlebrook/OADC, in line with the optimized formulation of Middlebrook media for mycobacterial growth and dispersion. OADC supplementation includes components such as albumin and oleic acid, which may stabilize the mycobacterial cell surface and reduce lipid-driven aggregation. Mycobacteria are known to form aggregates due to their highly hydrophobic cell envelope, and previous studies have shown that detergents such as Tween 80 can reduce clumping and yield more homogeneous cultures [24]. Tween 80 was intentionally omitted here, as it may alter cell wall composition, bacterial physiology, and phage-host interactions, potentially affecting adsorption, plaque formation, and experimental reproducibility [25]. Clumping assays were therefore restricted to *M. smegmatis* and *M. abscessus* subsp. *abscessus*, as aggregation in the other tested isolates was minimal and readily resolved by vortexing.

Phage activity across hosts, media, and assay formats was summarized using categorical heat maps (Fig. 3), distinguishing productive infections with countable plaques from lysis zones and complete absence of detectable activity. Spot assays were employed to accommodate the large experimental scale; notably, each condition was tested with six biological replicates and two technical replicates, representing a major strength of this study. However, the frequent observation of confluent lysis or lysis without countable plaques highlights an important limitation of spot assays for quantitative applications, as reliable PFU determination is not possible under these conditions. Nevertheless, such lysis patterns remain highly informative for rapid host-range screening and comparative assessment of cultivation and plating strategies. In this context, the heat-map approach enables efficient identification of permissive phage-host combinations that merit subsequent quantitative plaque assays, which remain the gold standard for PFU determination [26]. Furthermore, the absence of growth under certain conditions likely reflects strain-specific growth requirements and agar compatibility. The inclusion of this observation provides important context for defining practical limitations of each protocol and informs recommendations regarding approach selection.

A central finding of this work is the influence of assay format on phage detectability and reproducibility. While both, the top agar method and direct plating, supported phage-induced lysis, the top agar method consistently yielded a higher proportion of conditions with countable plaques across media and host species. This advantage was particularly evident in slow-growing NTM, whereas fast-growing species such as *M. smegmatis* showed more comparable performance between methods. In contrast, direct plating more often resulted in confluent lysis without discrete plaque formation, limiting its quantitative resolution. These differences are likely attributed to improved spatial confinement and enhanced local phage-host interactions in the semi-solid matrix of the top agar system, which facilitate plaque development [27]. In addition, technician-dependent and experimental variability (e.g., differences in plating technique, timing, or handling of bacterial suspensions) may have contributed to some of the observed differences, although the use of multiple biological and technical replicates aimed to minimize such effects; notably, the improved reproducibility observed with the top agar method may help mitigate variability in routine applications.

Quantitative analysis of conditions yielding countable plaques (Fig. 4) further demonstrated that the assay format primarily affected the kinetics and consistency of detectable phage activity rather than total PFU output. The top agar method generally resulted in earlier times to positivity and narrower PFU distributions, indicating more synchronous infection dynamics. Importantly, when both methods yielded countable plaques, total PFU values were comparable, underscoring that differences between protocols predominantly influence detection reliability rather than phage replication capacity.

Comparison of TSB/Glycerol and TSB/Yeast-based protocols did not reveal a consistent advantage of either formulation. Although glycerol has been reported to enhance plaque visibility and size in certain phage systems [28], plaque morphology was not a primary endpoint of this study. While glycerol is widely used in mycobacterial culture media, direct evidence linking glycerol supplementation to increased clumping in liquid culture remains limited and warrants further investigation.

Other media, such as Kirchner-based formulations and Löwenstein-Jensen agar, were excluded from this study due to their limited comparability with the experimental setup and their minimal previous application in phage/NTM assays. Kirchner medium contains horse serum, introducing undefined components and potential batch variability, which complicates controlled side-by-side analyses. Löwenstein-Jensen medium, a slope-based egg formulation, would require substantial adaptation of the assay workflow, further limiting direct comparability. Nevertheless, exploring the use of non-standardized media in NTM/phage studies could provide valuable insights into the suitability and reliability of these media for spot and plaque assays in more complex and clinically relevant culture systems.

In summary, the key findings of this study are: (i) all tested media support NTM liquid cultivation; (ii) bacterial clumping is least pronounced in Middlebrook/OADC; (iii) the top agar method is more robust and reproducible than direct plating for quantitative phage assays; and (iv) Middlebrook/OADC-based protocols incur higher costs and require slightly increased hands-on time. Researchers should therefore tailor protocol selection to experimental goals as well as to NTM species, balancing robustness, throughput, and cost efficiency. These conclusions are based on the specific set of isolates and mycobacteriophages investigated and should therefore be interpreted with caution; as this study does not include a systematic comparison of laboratory/reference strains and a broader panel of clinical isolates, further validation will be required to determine how well these findings capture the diversity encountered in clinical practice. For applications restricted to fast-growing NTM, TSB-based protocols may represent a cost-effective alternative for fast growing NTM, whereas studies involving slow-growing species benefit from Middlebrook/OADC-based approaches.

## Conclusion

This study delivers a comprehensive evaluation of variables relevant to *in vitro* phage testing, validated by multiple biological replicates and two distinct mycobacteriophages. Middlebrook/OADC-based protocols combine several advantages, including reliable cultivation of most tested NTM species and reduced aggregation in liquid culture, albeit at higher cost. TSB-based methods are more economical but were less reliable, particularly for slow-growing NTMs. Given the substantial heterogeneity in phage infection profiles among clinical NTM isolates and the resulting need for personalized therapeutic approaches, standardization remains challenging. Nevertheless, the comparative framework presented here provides practical guidance for laboratory preparation and protocol selection in mycobacteriophage research.

## Supplementary Information

Supplementary Material 1.

## Data Availability

All datasets generated and/or analysed during the current study are publicly available in the Zenodo repository at https://doi.org/10.5281/zenodo.18737049.

## Abbreviations

CFU: Colony Forming Units
DP: Direct Plating
mL: Milliliter
MAC: *Mycobacterium **avium* Complex
NTM: Nontuberculous mycobacterial
OADC: Oleic Albumin Dextrose Catalase
OD_600_: Optical Density at 600 nm
PBS: Phosphate Buffered Saline
PFU: Plaque Forming Units
TA: Top Agar
TSB: Tryptic Soy Broth
