# Supplementary material for: Targeting nontuberculous mycobacteria with phages: optimization of in vitro host range assays
Source: BMC Microbiol. 2026 Jul 30;26:682. doi: 10.1186/s12866-026-05452-3 (PMC13422341; doi:10.1186/s12866-026-05452-3)
Supplement: Supplementary file 1 — Supplementary Material 1. [file 12866_2026_5452_MOESM1_ESM.docx]

***Methods: Statistical Analysis Files (Zenodo Repository)***

Detailed information on the statistical analyses has been deposited on Zenodo (open-access research repository) and can be accessed via the following DOI: 10.5281/zenodo.18737049. All procedures were conducted in accordance with relevant guidelines and regulations.

***Methods: Bacterial strains and cultivation***


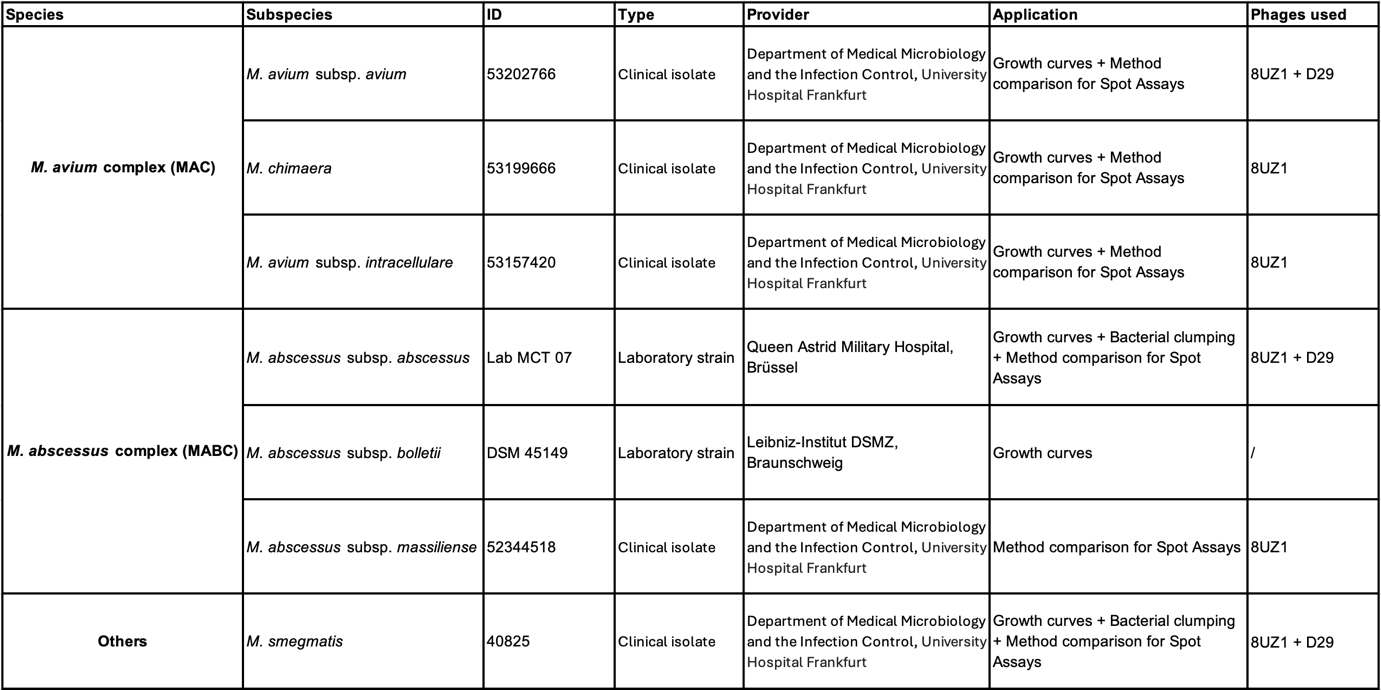


**Table S1.** Characteristics and experimental applications of the mycobacterial strains used in this study.

***Results: Titer-dependent detection of lytic activity of phage 8UZl***


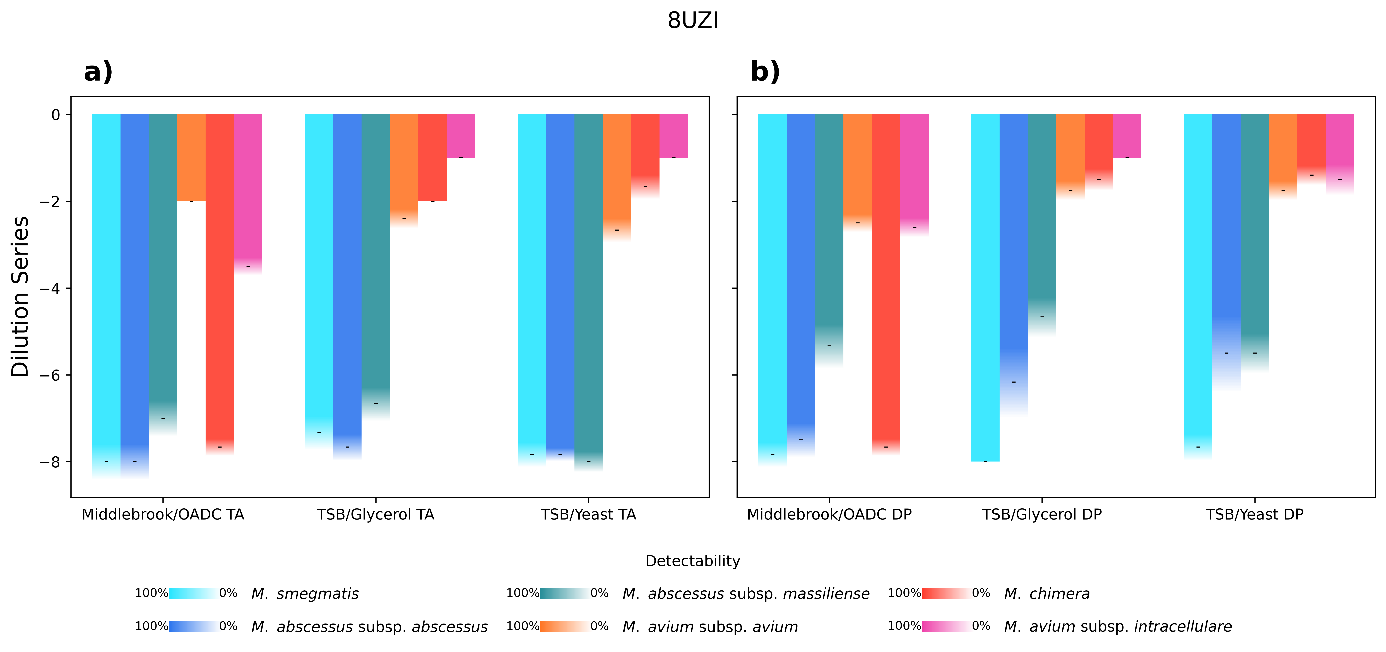


**Figure S1.** For all three methods (Middlebrook/OADC, TSB/Glycerol, and TSB/Yeast), bars denote the range of dilution at which 8UZl produced detectable lysis (lysis zones and/or countable plaques) in the bacterial lawn. Initial titer of 8UZl = 10^10^ PFU/mL. Experiments using the TA method are shown on the left (a), and those using DP are shown on the right (b). Expectation values represent the mean dilution factor at which the detection threshold was reached across all evaluable technical replicates per biological replicate and are indicated by a small dash; faded regions indicate the corresponding standard errors.

***Results: Validation in Rapidly Growing Mycobacteria Using Phage D29***


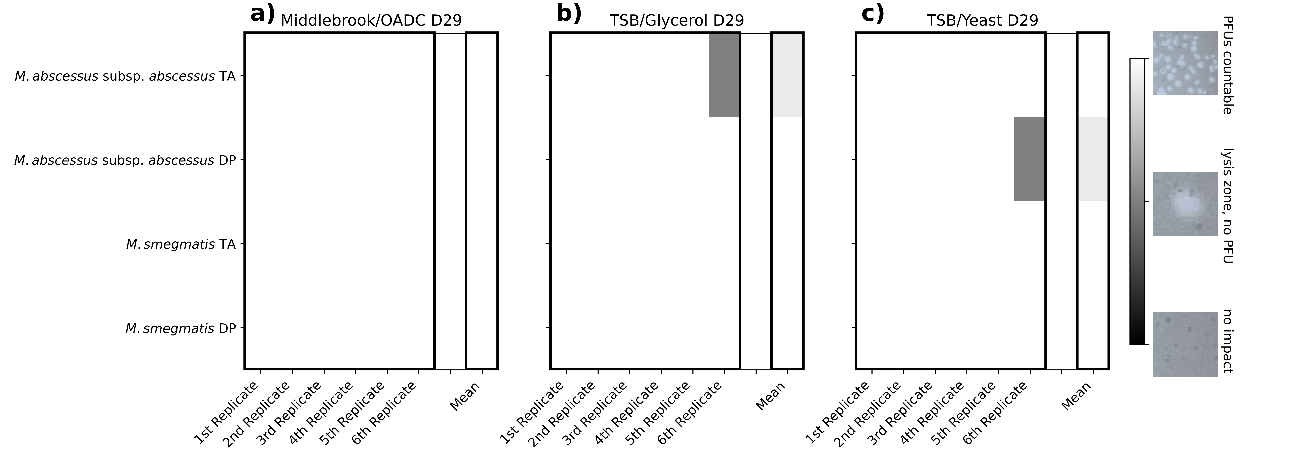


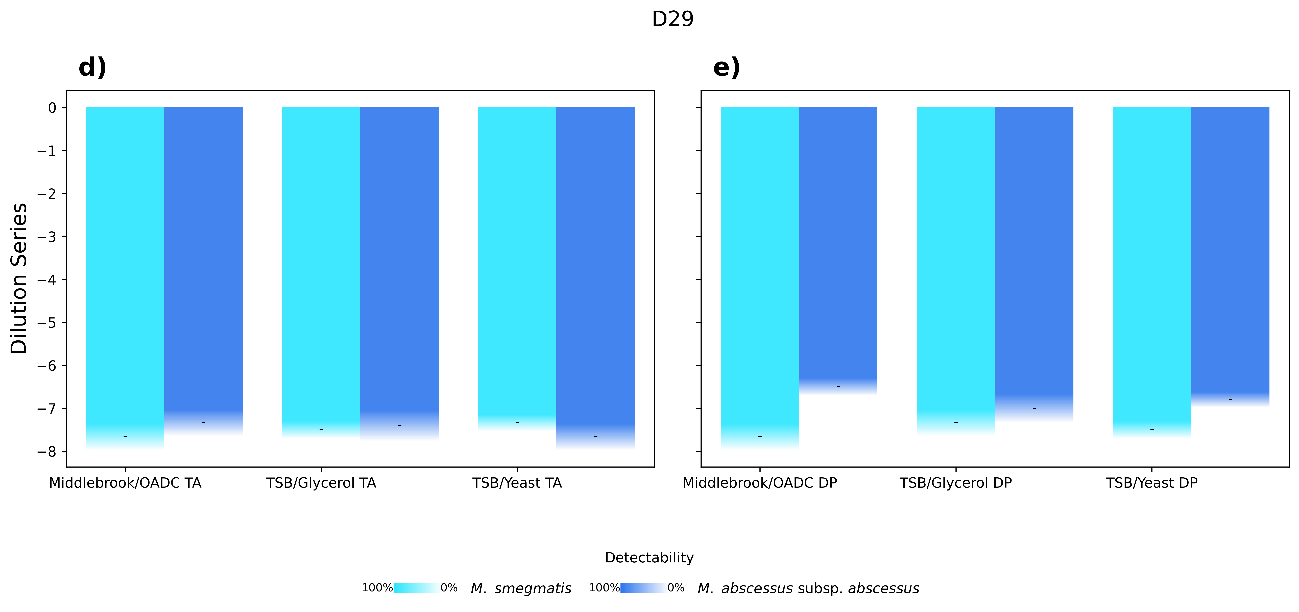


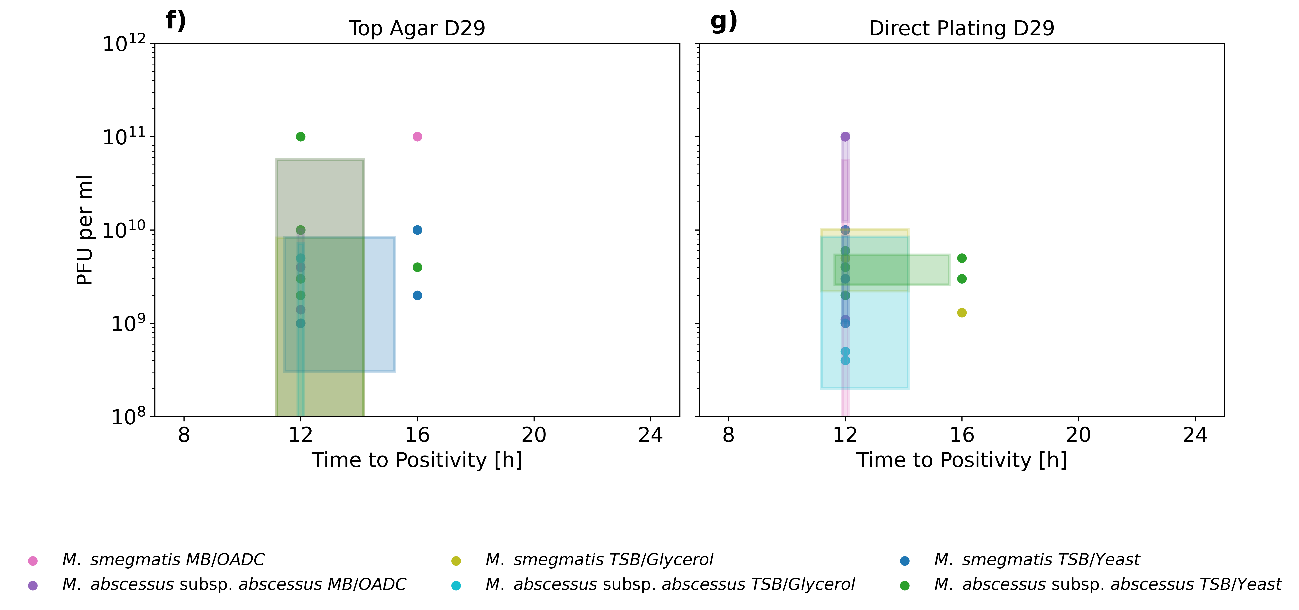


**Figure S2** Summary of data for D29 (initial titer of 10^10^), as previously shown for 8UZ1 in Figures 3–4. (a-c) Heat maps of D29 across bacterial strains with corresponding host range; (d) D29 impact in dilution series; and (e+f) plaque-forming units of D29 per milliliter. Note that the bacterial color schemes of e and f do not correspond to the images above, owing to the diversity of data points presented.
